# Supplementary material for: The electrical heart axis of the fetus between 18 and 24 weeks of gestation: A cohort study
Source: PLoS One. 2021 Dec 16;16(12):e0256115. doi: 10.1371/journal.pone.0256115 (PMC8675734; doi:10.1371/journal.pone.0256115)
Supplement: S1 File — (DOCX) [file pone.0256115.s002.docx]

RESEARCH PROTOCOL

Pilot study:

**Evaluation of the non-invasive fetal electrocardiogram, regarding the diagnosis of Congenital Heart Diseases.**

**PROTOCOL TITLE *Pilot study: Diagnosis of congenital heart disease with fetal ECG***

| **Protocol ID** | **Not applicable** |
| --- | --- |
| **NL number** | **48535.015.14** |
| **Short title** | **Pilot study: Diagnosis of congenital heart disease with fetal ECG** |
| **Dutch, simplified title** | **Pilot studie: Opsporen van congenitale hartafwijkingen middels foetaal ECG.** |
| **Date** | **17.12.2015** |
| **Principal investigator** | **Prof. Dr. S.G. Oei, gynecologist Máxima Medical Center Veldhoven**  **De Run 4600, 5504 DB Veldhoven**  **e-mail:** [**guidoei@outlook.com**](mailto:guidoei@outlook.com)  **Phone (office): +31 40 8888384** |
| **Multicenter research: per site** | **University Medical Center Utrecht (UMCU)**  **Investigator: dr. A. Kwee**  **Email: a.kwee@umcutrecht.nl**  **Radboud Medical Center Nijmegen (UMCN)**  **Investigator: Prof. Dr. F. Vandenbussche**  **Email: f.vandenbussche@obgyn.umcn.nl** |
| **Fellow investigator(s) (in Dutch: hoofdonderzoeker/ uitvoerder)** | **N.B. Eijsvoogel, intern obstetrics/gynecology**  **Drs. K.M.J. Verdurmen, resident**  **obstetrics/gynecology**  **Dr. J.O.E.H. van Laar, gynecologist**  **Máxima Medical Center Veldhoven**  **De Run 4600, 5504 DB Veldhoven**  **e-mail: kimverdurmen@live.nl**  **e-mail:** [**j.vanlaar@mmc.nl**](mailto:j.vanlaar@mmc.nl) |
| **Sponsor (in Dutch) :**  **verrichter/opdrachtgever)** | **Máxima Medical Center, Board of Management**  **Board of Management Máxima Medical Center (in Dutch: Raad van Bestuur)** |
| **Independent expert (s)** | **Dr P. Andriessen, pediatrician**  **Máxima Medical Center Veldhoven**  **De Run 4600, 5504 DB Veldhoven**  **e-mail: p.andriessen@mmc.nl** |

**PROTOCOL SIGNATURE SHEET**

| **Name** | **Signature** | **Date** |
| --- | --- | --- |
| **Coordinating Investigator/ /Principal investigator: Prof. Dr. S. G. Oei, gynecologist Máxima Medical Center** | 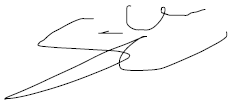 | **07-05-2014** |

**TABLE OF CONTENTS**

1. INTRODUCTION AND RATIONALE 9

2. OBJECTIVES 11

3. STUDY DESIGN 12

4. STUDY POPULATION 14

4.1 Population (base) 14

4.2 Inclusion criteria 14

4.3 Exclusion criteria 14

4.4 Sample size calculation 15

5. TREATMENT OF SUBJECTS 16

5.1 Investigational product 16

6. METHODS 17

6.1 Study parameters/endpoints 17

6.1.1 Main study parameter/endpoint 17

6.1.2 Secondary study parameters/endpoints (if applicable) 17

6.2 Randomization, blinding and treatment allocation 17

6.3 Study procedures 17

6.4 Withdrawal of individual subjects 18

7. SAFETY REPORTING 19

7.1 Section 10 WMO event 19

7.2 AEs and SAEs 19

7.2.1. Adverse events (AEs) 19

7.2.2. Serious adverse events (SAEs) 19

7.3 Reporting adverse events 19

7.4 Follow-up of adverse events 20

8. STATISTICAL ANALYSIS 21

8.1 Primary study parameter(s) 21

8.2 Secondary study parameter(s) 21

9. ETHICAL CONSIDERATIONS 22

9.1 Regulation statement 22

9.2 Recruitment and consent 22

9.3 Benefits and risks assessment 22

9.4 Compensation for injury 23

10. ADMINISTRATIVE ASPECTS, MONITORING AND PUBLICATION 24

10.1 Handling and storage of data and documents 24

10.2 Monitoring and Quality Assurance 24

10.3 Amendments 24

10.4 Annual progress report 24

10.5 End of study report 24

10.6 Public disclosure and publication policy 25

11. STRUCTURED RISK ANALYSIS 26

11.1 Potential issues of concern 26

12. REFERENCES 27

13. APPENDIX I 29

14. APPENDIX II 30

**LIST OF ABBREVIATIONS AND RELEVANT DEFINITIONS**

| **ABR** | **ABR form, General Assessment and Registration form, is the application form that is required for submission to the accredited Ethics Committee (In Dutch, ABR = Algemene Beoordeling en Registratie)** |
| --- | --- |
| **AE** | **Adverse Event** |
| **AR** | **Adverse Reaction** |
| **CA** | **Competent Authority** |
| **CCMO**  **CHD** | **Central Committee on Research Involving Human Subjects; in Dutch: Centrale Commissie Mensgebonden Onderzoek**  **Congenital heart disease** |
| **CV** | **Curriculum Vitae** |
| **DSMB**  **fECG** | **Data Safety Monitoring Board**  **Fetal Electrocardiogram (in Dutch: foetaal electrocardiogram)** |
| **GCP** | **Good Clinical Practice** |
| **GUO IC** | **Advanced ultrasound research. In Dutch: Geavanceerd ultrageluid onderzoek Informed Consent** |
| **METC**  **MMC**  **SD**  **SEO** | **Medical research ethics committee (MREC); in Dutch: medisch ethische toetsing commissie (METC)**  **Máxima Medical Center**  **Standard deviation**  **Structural ultrasound research (in Dutch: structureel echografisch onderzoek)** |
| **SPC** | **Summary of Product Characteristics (in Dutch: officiële productinfomatie IB1-tekst)** |
| **Sponsor** | **The sponsor is the party that commissions the organisation or performance of the research, for example a pharmaceutical**  **company, academic hospital, scientific organisation or investigator. A party that provides funding for a study but does not commission it is not regarded as the sponsor, but referred to as a subsidizing party.** |
| **Wbp** | **Personal Data Protection Act (in Dutch: Wet Bescherming Persoonsgevens)** |
| **WMO** | **Medical Research Involving Human Subjects Act (in Dutch: Wet Medisch-wetenschappelijk Onderzoek met Mensen** |

**SUMMARY**

**Rationale:** Congenital heart disease (CHD) is a severe condition, which needs early detection and treatment. The current method for detecting CHD during pregnancy is a structural ultrasound around week 20 of gestational age. Only 25 to 60 per cent of the cases are detected by this method. Therefore, there is need for a technique with a higher sensitivity, in order to guarantee early detection. This new technique could be the transabdominal non-invasive fetal electrocardiogram (fECG). In order to detect the different abnormalities, the normal ranges of amplitudes and segment intervals of the fECG have to be established.

**Objective**: To detect the normal range of amplitudes, segment intervals and the heart axis of the fECG. To compare fECG of healthy fetuses and fetuses with severe CHD.

**Study design:** This study will be performed as a cross-sectional and a case-cohort study. The first part of the research (cross-sectional study) focuses on the normal range of amplitudes and segment intervals of the fECG. The second part (case-cohort study) will focus on the values of the amplitudes and segment intervals of fetuses with diagnosed a severe CHD like Fallot’s tetralogy. CHD is diagnosed by the current method for prenatal screening, the structural ultrasound. The center, at which the CHD is diagnosed, informs the patient about our study and contacts us if the patient is willing to participate in the study. Centers involved in this research are the tertiary care hospitals: Máxima Medical Center Veldhoven (MMC), University Medical Center Utrecht (UMCU), Radboud Medical Center Nijmegen (UMCN), Acadamic Medical Center Amsterdam (AMC).

**Study population:** In the cross-sectional study, 200 pregnant patients, aged older than 18 years, with a gestational age of 18 – 24 weeks will be included. The fetuses have to be healthy, without any known congenital heart abnormalities. For the case-cohort study, the fetus must be diagnosed with a severe, hemodynamic important CHD. We will include 10 patients with the same sort of severe CHD. The types of CHD we will include are: Fallot’s Tetralogy, hypoplastic left heart syndrome, aortic stenosis, pulmonary atresia, transposition of the great vessels, coartcation of the aorta and an atrial ventricular septal defect

**Intervention**: The fECG is a non-invasive, transabdominal approach with self-adhesive electrodes. The recordings are performed between 08.00 h and 16.00 h during appointments at the outpatient clinic and will take no longer than 45 minutes. The patient will be lying on a comfortable bed in a semi-recumbent position.

**Main study parameters/endpoints:** to determine the normal values and ranges of amplitudes, segment intervals and heart axis of healthy fetuses with a gestational age of 18 to 24 weeks. To establish the differences in fECG between healthy fetuses and fetuses diagnosed with severe CHD.

**Nature and extent of the burden and risks associated with participation, benefit and group relatedness:** There is no risk associated with the recordings of the fECG. There might be some minor skin irritation caused by the self-adhesive electrodes. Measurements are combined with regular hospital visits.

# 1. INTRODUCTION AND RATIONALE

During pregnancy, multiple ultrasound examinations are performed to assess the fetal condition. This is executed, amongst others, during or around week 20 of gestational age and is called the fetal anomaly ultrasound (SEO in Dutch). With this technique, all kind of possible congenital anomalies can be detected, including CHD. However, a false negative diagnosis occurs frequently and the SEO only detects 25 to 60 per cent of the patients with CHD^1, 2^. That makes CHD the most common structural fetal abnormality of which a significant part is missed during the prenatal life.

CHD can be divided into cyanotic and non-cyanotic. An example of cyanotic CHD is Fallot’s tetralogy or a transposition of the great vessels. An example of the non-cyanotic form is an aortic stenosis, atrial septum defect or an aortic coarctation. The incidence of CHD is estimated to be 4 to 9 per 1000 live born^3^. In the Netherlands, the overall incidence of anomalies of the vascular system is estimated at 6,6 per 1000 live births^4^. The incidence of severe CHD (read, of hemodynamic importance) is low (see appendix I. page 27).

The survival for patients with CHD is low, 21 percent^2, 3^, and the prognosis is negatively influenced by the presence of heart failure, aneuploidy or extracardial malformations. There are several studies which show a correlation between an abnormal heartbeat of the fetus and the diagnosis CHD. These patterns can be diagnosed with ultrasound (including the Doppler technique), which is the current method for prenatal screening of CHD. If a CHD is suspected or seen on the SEO, the patient will receive a more intensive ultrasound, named the GUO (geavanceerd ultrageluids onderzoek) in Dutch. If the CHD is confirmed with the GUO, the fetus has a high certainty of having a CHD. In 2003, the Dutch magazine of medicine (in Dutch: Nederlands tijdschrift voor geneeskunde) reported a sensitivity of 94,7%, a specificity of 98,8%, a positive prognostic value of 95,3% and a negative prognostic value of 98,6%^5^

However, these routine controls with ultrasound can only diagnose about 25 to 60 per cent of the large heart defects^1, 2^. The disadvantage of Doppler ultrasound is that there can be false – negatives. Plus the fact that Doppler can’t detect beat – to – beat heart rate, which causes several heart abnormalities to be missed.

The medical world needs a reliable non-invasive diagnostic method with a better detection rate of CHD. However, up till now, a transabdominal non-invasive fECG was too difficult to accomplish. Velayo et al^2^ was the first to report the clinical possibility of using fECG to detect CHD in 2011. They conducted a prospective study with a total of 179 women with singleton pregnancies from 18 to 41 weeks of gestation. All patients received an ultrasound investigation and a fECG on the same day. They found a sensitivity of 100% and a specificity of 99% of the fECG for detecting CHD. It is possible that after combining the fECG with the ultrasound, the sensitivity and specificity will be even higher.

Most forms of CHD have an abnormal fECG^5^. The most important aspect of the ECG is the heart axis, for example right axis deviation. This is seen in most of the severe CHD forms, like Fallot’s tetralogy. Other possible abnormal aspects of the fECG are amplitudes and segment intervals (prolonged or premature).

Researchers of the Máxima Medical Center (MMC) and the Eindhoven University of Technology are working in collaboration to develop the fECG^1,6^. The fECG can be performed non-invasively during pregnancy^7^. Like the fetal anomaly ultrasound, it can be used from 18 weeks of gestational age onwards. As stated above, CHD will most likely be marked by fECG changes^5^.

The first difficulty of conducting a fECG is that the fetus is surrounded by amniotic fluid and maternal tissues, which enlarges the distance to the electrodes. Second, the fetus moves around, which makes it difficult to measure the heart from one single direction. On top of that, at a gestational age of 20 weeks the fetal heart is about 1/10^th^ of the size of an adult heart^6^.

The fECG during pregnancy is difficult to interpret. The main reason for this is a physiological dominance of the fetal right ventricle during pregnancy. This is caused by the circulation of the fetus in utero. Thus, right-sided obstructive lesions such as Fallot’s tetralogy or pulmonary hypertension with a dominance of the right ventricle are difficult to diagnose in utero. These right-sided obstructive lesions are usually accompanied by left-sided obstructive lesions such as aortic stenosis or coarctation of the aortae^8^. Because of the difficulty of diagnosing right-sided obstructive lesions, the lesions combined with left-sided obstruction can be detected more easily.

We expect that the fECG can detect severe heart conditions early in pregnancy, with the possibility of adequate early treatment in the future. Nowadays, there are options of operating during pregnancy to optimize the outcome of the fetus with CHD.

We hypothesize that the non-invasive fECG combined with the SEO has a higher detection rate for diagnosing CHD compared to the SEO alone. We expect that the fECG can detect additional details about the development and etiology of CHD. More knowledge about fetal heart rhythms and abnormalities of the different intervals and heart axis in a fECG are needed to optimize the diagnosis and management of CHD.

The summary of the most occurring ECG findings of the most common CHD can be found in Appendix II, page 28.

# 2. OBJECTIVES

Primary Objective: To establish the normal ranges or values of amplitudes, segment intervals (with 95% confidence intervals) and the heart axis of the fECG in a healthy fetus.

Secondary Objective: To compare the fECG in various forms of severe CHD with the “normal” fECG. To determine the diagnostic value of fECG to detect CHD.

# 3. STUDY DESIGN

Both the cross-sectional and case-cohort study are prospective in nature. In the future, a longitudinal cross-sectional study can be performed based upon this study.

The cross-sectional study will take place in the MMC, Veldhoven, the Netherlands and Diagnostic center Eindhoven, the Netherlands. All the patients who visit the outpatient clinic for the first time will receive information about the research. Patients who visit the Diagnostic center Eindhoven will receive information about the research via their appointment confirmation e-mail. Measurements will be performed before or after the SEO or GUO-1 after written informed consent; patients do not have to come to the hospital or the diagnostic center for an extra visit.

The case-cohort study will take place in the MMC Veldhoven, and the medical centers Radboud MC Nijmegen, Academic Medical Center Amsterdam, and Maastricht UMC the Netherlands. Whenever a patient is diagnosed with a fetal severe CHD in any of these centers by using the SEO and the GUO, they will inform the patient about our research and contact us after informed consent. We will perform a fECG during routine follow up visits in those centers. For more information on recruiting the patients, see chapter 9, paragraph 9.2 “Recruitment and consent”.

In the cross-sectional study, 200 pregnant women will be included. With this amount of patients, we can determine with the wanted accuracy what the normal values and 95% CI of a healthy fetus are^9, 10^. If any of these fetuses is diagnosed with a CHD, they will automatically be evaluated in the case-cohort study.

In the case-cohort study, we will include pregnant women carrying fetuses with a form of severe CHD, preferably with the same CHD. A severe CHD is defined as possibly life threatening and/or needing intervention or surgery in the first year of life. For now, we believe that of every CHD, we need 10 patients to determine the difference between the normal values and the segment intervals of the fetuses with this severe CHD. The overall incidence of CHD is 4 to 9 per 1000 living newborns^4^. Because of this low incidence, it would take years to include more than 10 fetuses per form of severe CHD. Because of this reason, plus the fact this concerns a pilot study, we chose to include 10 fetuses per form of severe CHD. Because of this low incidence, collaboration with more than one center is needed. We believe that we will be able to collect data of the fECG of 10 fetuses with the support of the other centers.

The fECG signals are stored and digitized by a program named the NEMO system^1^. More details can be found in chapter 4.

With the collected data, we will perform several sub-analyses. We will calculate the normal values and ranges of the amplitudes (P, QRS, T), and the segment intervals with 95% confidence intervals. We will also evaluate the heart axis of the fetus. This data will be compared with the collected data of the fetuses with CHD. The patients will not be notified of any abnormal findings on the fECG, because this contains a pilot study and any findings on the fECG are still being evaluated. Therefore, this research does not have any consequences for the standard care plan of the patients and their fetuses.

If any of the fECG turns out to be abnormal, it will not influence the treatment of the fetus. The main reason for the last fact is that we do not know if the findings we think are abnormal are actually abnormal. This is one of the aspects we would like to investigate.

In the future, a longitudinal research is needed to determine the sensitivity and specificity of fECG.

Approximately 10 weeks postpartum, the patients from the cross-sectional study will receive a questionnaire about their delivery and their newborn and patient information can be withdrawn from the postnatal clinic or the attending pediatrician. If the participants do not return the questionnaire, they will be excluded from further analyses.

# STUDY POPULATION

## 4.1 Population (base)

In the cross-sectional study, 200 pregnant women carrying a healthy, singleton fetus with a gestational age between 18 and 24 weeks, who visit the outpatient clinic in MMC Veldhoven or Diagnostic center Eindhoven, will be included. If any of the fetuses turn out to have a form of CHD, the data will be either excluded or analyzed in the case-cohort study.

In the case-cohort study, pregnant woman carrying a singleton fetus, with a gestational age between 18 and 24 weeks, with a known severe CHD will be included. We will include patients with the following CHD: Fallot’s tetralogy (possible with pulmonary atresia of stenosis), hypoplastic left heart syndrome, aortic stenosis, transposition of the great vessels, coartcation of the aorta and atrial ventricular septal defect. We chose these types because they are severe and have hemodynamic consequences if they do not get the proper treatment. Because of the low incidence of these types of CHD (see appendix I page 27), we believe it is impossible to gather more than 10 patients per CHD. Therefore, we will include 10 patients in every CHD group, leading to a total of 60 patients with 6 different forms of severe CHD. We believe that we will include 20 patients per participating center, thus 2 forms of CHD in every center. We believe this will be enough to detect any big differences between the fECG of the healthy fetuses and the fECG of the fetuses with CHD, but we are aware that more research will be needed in the future. Because the incidence in our medical center is low, collaboration with more than one center is needed.

## Inclusion criteria

In order to be eligible to participate in the cross-sectional study, a subject must meet all of the following criteria:

- Pregnant women carrying a healthy fetus
- Aged older than 18 years
- Gestational age between 18 and 24 weeks

In the case-cohort study, a subject must meet all of the following criteria:

- Pregnant woman carrying a fetus with a known severe CHD (Fallot’s Tetralogy,

hypoplastic left heart syndrome, aortic stenosis, pulmonary atresia, transposition of the great vessels, coartcation of the aorta and an atrial ventricular septal defect)

- Aged older than 18 years
- Gestational age between 18 and 24 weeks

## Exclusion criteria

A potential subject who meets any of the following criteria will be excluded from participation in this study:

- Multiple Pregnancies
- Insufficient understanding of Dutch language

## Sample size calculation

This will be the first study to investigate the clinical contribution of fECG for the diagnosis of severe CHD. We believe that a total of 200 patients will be enough to evaluate the normal range or value of the different segment intervals and amplitudes of the fECG, as described in the literature by Douglas G. Altman^10^. For the case-cohort study, we believe that a total of 10 patients per severe CHD will be enough for the evaluation and the comparison of the fECG of a healthy fetus and the fECG of a fetus with known severe CHD. No sample size calculation was used in the estimation of the size of the population for the case-cohort study, since this is a pilot study.

# 5. TREATMENT OF SUBJECTS

The intervention used in this study will be the transabdominal, non-invasive fECG.

## Investigational product

The fECG is a non-invasive, transabdominal research method. It uses multiple electrodes on the maternal abdomen to determine the fetal heart rhythm and some parts of the heart’s electrical conduction system.

The pregnant women will be lying down on a comfortable bed in semi-recumbent position. The fECG is conducted with eight electrodes placed on the abdomen, in a fixed configuration. Before applying the electrodes on the abdomen, the skin will be scrubbed to optimize the impedance. On the right side of abdomen a ground electrode is placed.


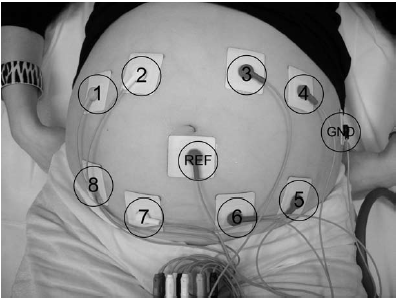


Figure 1: the configuration of the electrodes on the maternal abdomen^1^.

The eight electrodes give bipolar signals of which the fECG comprises. The placement of the electrodes is chosen in order to assess the fetal heart with as much accuracy as possible, as seen in figure 1. Because the fetus can move freely in the uterus, as large as possible number of ECG signals/electrodes is preferred. With eight electrodes, at least some of the electrodes will be close by the fetal heart and thus will give a usable bipolar signal. With this amount of electrodes, we will be able to detect the bipolar signals of the fetal heart in any circumstance. The fECG consists of a 12 – lead signal.

As mentioned in chapter 2, the ECG signals are stored and digitized by a system named the NEMO system, which comprises of a programmable amplifier for the acquiring of electrophysiological signals and a PC for the controlling of the settings of the amplifier and storage of the recordings. This amplifier is based on the M-PAQ (Maastricht Instruments BV, the Netherlands) and modified to maximize the performance^1^.

# 6. METHODS

## Study parameters/endpoints

### Main study parameter/endpoint

In the cross-sectional study, the main study parameter is the determination of the normal value or range of amplitude and segment intervals with 95% confidence intervals of the fECG of a healthy fetus.

### Secondary study parameters/endpoints (if applicable)

The case-cohort study focuses on the abnormal value or range of amplitude and segment intervals in fetuses with diagnosed severe CHD, and compares these with the values found in the cross-sectional study.

## Randomization, blinding and treatment allocation

There will be no randomization or blinding.

## Study procedures

All the patients will undergo a non – invasive, transabdominal fECG during regular outpatient clinic appointments, performed by one of the researchers. This test has a very low grade of discomfort for the patient. The conducted fECG will be stored on an external hard drive, the data will be analyzed by researchers of the Technical University. The last two investigators will look at the fECG and will determine the segment intervals, the heart axis and the amplitudes. After the analysis, we will be able to enter the data in SPSS to conduct normal values and ranges with 95% confidence intervals. The patients will not be notified of any abnormal findings on the fECG, because this is a pilot study. We do not know for certain that any abnormalities we find are actually real abnormalities. This is something we want to investigate.

In the cross-sectional study, we want to know if the fetuses we included are born healthy. Therefore, we will evaluate this with a simple questionnaire which we will send four to six weeks postpartum. We believe this questionnaire will be sufficient to evaluate if any severe CHD was present at birth, because a severe CHD has hemodynamic consequences which present early in neonatal life. Besides, we have permission to withdraw patient information at the postnatal clinic or attending pediatrician. If the neonate has a congenital heart disease missed by the SEO, we will exclude the patient from the cross-sectional study and will evaluate the data of the fECG in the case-cohort study. If the participants do not return the questionnaire, they will be excluded from further analyses.

## Withdrawal of individual subjects

If subjects do not want to proceed with the measurement, they can step out at any moment. There are no follow – up moments, which makes it easier for the patients to join the research.

# 7. SAFETY REPORTING

## Section 10 WMO event

In accordance to section 10, subsection 1, of the WMO, the investigator will inform the subjects and the reviewing accredited METC if anything occurs, on the basis of which it appears that the disadvantages of participation may be significantly greater than was foreseen in the research proposal. The study will be suspended pending further review by the accredited METC, except insofar as suspension would jeopardize the subjects’ health. The investigator will take care that all subjects are kept informed.

## 7.2 AEs and SAEs

### 7.2.1. Adverse events (AEs)

## We don’t expect any adverse events during the study, however when applying the electrodes on the skin, an allergic reaction can exist. This is rare.

### 7.2.2. Serious adverse events (SAEs)

A serious adverse event is any untoward medical occurrence or effect that:

- results in death;
- is life threatening (at the time of the event);
- requires hospitalization or prolongation of existing inpatients’

hospitalization;

- results in persistent or significant disability or incapacity;
- is a congenital anomaly or birth defect;

Any other important medical event that may not result in death, be life threatening, or require hospitalization, may be considered a serious adverse experience when, based upon appropriate medical judgment, the event may jeopardize the subject or may require an intervention to prevent one of the outcomes listed above.

We do not expect SAEs during the study due to the non-invasive technique we use, but if any SAE occurs we will report them and, if necessary, the patient will be followed by a medical doctor.

## Reporting adverse events

If an allergic reaction exists, we will report this as an adverse event after the study has been closed. Other (unexpected) SAEs, will be reported directly and these patients will be followed by a medical doctor, if this is necessary. The sponsor will report the unexpected SAEs through the web portal *ToetsingOnline* to the accredited METC that approved the protocol, within 15 days after the sponsor has first knowledge of the serious adverse reactions. SAEs that result in death or are life threatening should be reported expedited. The expedited reporting will occur not later than 7 days after the responsible investigator has first knowledge of the adverse reaction. This is for a preliminary report with another 8 days for completion of the report

## Follow-up of adverse events

All AEs will be followed until they have abated, or until a stable situation has been reached. Depending on the event, follow up may require additional tests or medical procedures as indicated, and/or referral to the general physician or a medical specialist.

# 8. STATISTICAL ANALYSIS

The collected data will be analyzed through SPSS 19 and two different outcome values will be calculated. The different outcome values are:

- Mean and standard deviation of segment intervals (PQ, QRS, ST etc.) with 95%

confidence intervals

- Mean and standard deviation of amplitudes of the P – top, QRS complex and the

T – top with 95% confidence intervals.

- The heart axis of the fetus

After analysis of these sub-groups (fetuses with the same form of severe CHD in one group, all the healthy fetuses in one group) in SPSS, the different intervals of the fECG of the cross-sectional study and the case-cohort study will be compared with one another through a t-test or a Mann-Whitney-Wilcoxon-test to investigate whether or not there is a repetitive pattern to be recognized. This depends from the normal distribution and the quantity of the collected data. The research is quantitative.

The questionnaires will be analyzed. We will evaluate per fetus if the conducted data from the cross-sectional population is usable and if the data of this particular patient should be excluded due to a missed diagnosis of congenital heart disease during the pregnancy.

## Primary study parameter(s)

The mean and standard deviation of the segment intervals and the amplitudes (with 95% confidence intervals) and the heart axis of healthy fetuses without known CHD.

## Secondary study parameter(s)

The typical patterns of the fECG of fetuses with a known severe CHD.

#

#

# ETHICAL CONSIDERATIONS

## Regulation statement

This study will be conducted according to the principles of the Declaration of Helsinki (64^th^ WMA General Assembly, Fortaleza, Brazil, October 2013) and in accordance with the Medical Research Involving Human Subjects Act (WMO).

## 9.2 Recruitment and consent

The patients of the cross-sectional study will be recruited through the gynecology outpatient clinic and through the Diagnostic center Eindhoven. The patients will receive information about the research during the appointment at the outpatient clinic, when the SEO or GUO-1 will be planned or via e-mail with the confirmation of their appointment for a SEO at the Diagnostic center Eindhoven. This information contains all the aspects and details of the research. Attached to the patient information letter, the patients will find an informed consent form. If patients have any questions, one of the researchers will answer these questions. Before the fECG will be conducted, the researcher will explain the meaning of the research again. The time of consideration will vary between different patients.

The patients of the case-cohort study will be recruited after the SEO and they will have recently discovered that their baby has a form of severe CHD. The follow up will be provided by a gynecologist at MMC Veldhoven, Radboud MC Nijmegen, AMC Amsterdam or Maastricht UMC. These specialists will ask the patients if they want to join the research. They will receive the information letter. Attached to the patient information letter, the patients will find an informed consent form. If wanted, the researcher can provide some extra information before conducting the fECG. It is important to conduct the fECG before week 24. The time of consideration will vary between different patients.

## 9.3 Benefits and risks assessment

No risks or adverse effects for the patient, the fetus or third parties are to be expected, except for a small chance of skin irritation due to adhesion of the electrodes to the skin. The electrodes used are known to be safe, since these electrodes are used in adult cardiology electrocardiograms. The electrodes are applied to the patient’s skin. The electrodes will transmit the electrical signal from the fetus heart to the processor^1^. In prior research concerning the non-invasive fECG, it was seen that the electrodes are safe^1, 6, 7^. All the used equipment is approved by the Medical Technical Service Department of the MMC in safety tests.

Because of the risk of aortocaval compression, the patient will be lying in a semi-recumbent position or a left lateral tilt position during the measurements. Aortocaval compression occurs because of the enlarged uterus compressing both the inferior vena cava and the lower aorta when lying in supine position. This will be prevented by the positions as mentioned above.

The benefits of this study are explained in chapter 1. In summary, the fECG will be used combined with the SEO to form a better diagnostic tool to diagnose severe CHD.

## 9.4 Compensation for injury

The sponsor/investigator has a liability insurance which is in accordance with article 7, subsection 6 of the WMO. As described above in paragraph 9.3, “benefits and risks assessment”, it is unlikely for any adverse advents to take place. The fECG with the non-invasive electrodes on the abdominal skin of the mother is a very safe manner of diagnosing fetal heart anomalies. In prior research, no adverse events were reported. Therefore, dispensation from statutory obligation to provide insurance was granted.

Because conducting the fECG will take more time than a regular appointment at the outpatient clinic, we will provide free parking cards for the patients.

**9.5 Independent expert**

The independent expert for our study will be dr. P. Andriessen, pediatrician in the Máxima Medical Center Veldhoven

# 10. ADMINISTRATIVE ASPECTS, MONITORING AND PUBLICATION

## 10.1 Handling and storage of data and documents

The data will be stored in a computer program named the NEMO system. This program can digitize and store the data of the fECG. The data will be coded, stored on a hard disk and will be handled with care. The patients will be numbered. Only the principal investigator will have the key to the code. The data will be kept for 15 years after termination of this study.

## 10.2 Monitoring and Quality Assurance

In this particular research, the principal investigator will monitor the conduct of the study. He will make sure that the data is kept safe and will ask for weekly updates. In case of any misunderstandings, he will be the contact person. Auditors and monitors from the Clinical Trial Centre Maastricht (CTCM) are able to evaluate measurements which have taken place in the MUMC. They are able to obtain data access only through the principal investigator and only if necessary.

## 10.3 Amendments

Amendments are changes made to the research after a favorable opinion by the accredited METC has been given. All amendments will be notified to the METC that gave a favorable opinion.

## 10.4 Annual progress report

The sponsor/investigator will submit a summary of the progress of the trial to the accredited METC once a year. Information will be provided on the date of inclusion of the first subject, numbers of subjects included and numbers of subjects that have completed the trial, serious adverse events/ serious adverse reactions, other problems, and amendments.

## 10.5 End of study report

The investigator will notify the accredited METC of the end of the study within a period of 8 weeks. The end of the study is defined as the last patient’s last visit.

In case the study is ended prematurely, the investigator will notify the accredited METC within 15 days, including the reasons for the premature termination.

Within one year after the end of the study, the investigator/sponsor will submit a final study report with the results of the study, including any publications/abstracts of the study, to the accredited METC.

## 10.6 Public disclosure and publication policy

Conform the “CCMO statement on publication policy”^11^, as well the positive as negative results will be published. Articles will be written by the principal investigators, mentioned on page 1, as well as other medical students and third parties participating in the study.

#

# 11. STRUCTURED RISK ANALYSIS

## 11.1 Potential issues of concern

*a. Level of knowledge about mechanism of action*

The fECG has been used for more research by Drs. K. Verdurmen, protocol ID nr. NL43294.015.13 and METC number 1307. There is enough knowledge about the fECG and there appear to be no known patho-physiological consequences for the mother and the fetus.

*b. Previous exposure of human beings with the test product(s) and/or products with a similar biological mechanism*

Mr. Ir. R. Vullings, post doc, has investigated this mechanism and has shown that the fECG is safe for patient use^1,6^. The fECG is also used for previously approved ongoing research by Drs. K. Verdurmen, protocol ID nr. NL43294.015.13 and METC number 1307.

*c. Study population*

The cross-sectional study requires healthy pregnant women. The physical condition of the patients is well enough to participate in the study. The case-cohort study requires healthy pregnant woman, but the fetuses have a known severe form of CHD. The condition of the mother AND the fetus will have to be stable enough to participate in the study.

If the mother is in a suboptimal condition, the fECG can still take place. Because the fECG is non-invasive with minimal mental pressure, it will not bring the patient in any risk. If in any case the fECG does bring a new diagnosis to the table, mental support will be offered.

# 12. REFERENCES

1. R. Vullings and S.G. Oei, “Can the 12 – lead ECG representation of fetal supraventricular extra systoles be used to assess congenital heart disease?” Chapter 2, Thesis
2. C. Velayo et al. *Understanding congenital heart defects through abdominal fetal electrocardiography: Case reports and clinical implications*. J. Obstet. Gynaecol. Res. Vol. 37, No 5: 428 – 435, May 2011
3. Tim van Mieghem, Philip DeKoninck, Patricia Steenhaut, Jan Deprest; *Methods for prenatal assessment of fetal cardiac function*. Prenat Diagn 2009; 29; 1193-1203.
4. Reefhuis J, Samrén EB, Diem MT van. Tables 1981 – 1998. *EUROCAT registration of congenital anomalies Northern Netherlands and Southwestern Netherlands*. Groningen: Univeristy of Groningen, 2000.
5. Wladimiroff J.W., Cohen-Overbeek TE, Ursem NTC, Bijma H, Los FJ; *Geavanceerd ultrageluidonderzoek naar aangeboren afwijkingen in Rotterdam; 20 jaar ervaring*. Ned Tijdschr Geneeskd 2003; 147 (43)
6. R. Vullings and S.G. Oei, “Can the 12 – lead ECG representation of fetal supraventricular extrasystoles be used to assess congenital heart disease?” Chapter 10, Thesis
7. van Laar JOEH, Warmerdam GJJ, Verdurmen KMJ, Vullings R, Peters CHL, Houterman S, et al. *Fetal heart rate variability during pregnancy, obtained from non-invasive electrocardiogram recordings*. Acta Obstet Gynecol Scand 2014; 93:93–101.
8. Gurleen Sharland; *Fetal cardiac screening and variation in prenatal detection rates of congenital heart disease: why bother with screening at all?* Future Cardiology, March 2012, Vol. 8, No. 2 , Pages 189-202
9. Johan Smits & Ronald Edens; *Onderzoek met SPSS en Excel*. Tweede druk. 2009 Pearson Education Benelux, Amsterdam.
10. Douglas G. Altman; *Practical statistics for medical research*. Taylor & Francis Ltd 1990
11. CCMO-statement publicatiebeleid. Maart 2002.
12. Myung K. Park, MD; Warren G. Guntheroth, MD. *How to read pediatric ECGs*. 4^th^ edition. 2006. Mosby , Inc,, an affiliate of Elsevier Inc. page 250-251
13. Ueda K, Ikeda T, Iwanaga N, et al. *Intrapartum fetal heart rate monitoring in cases of congenital heart disease*. Am J Obstet Gynaecol 2009; 201:64.e1-6
14. P.J. Sterk, K.F. Rabe; *The joy of writing a paper*. Breathe, march 8, volume 4, no. 3.

1. F. Hofmeyr et al. *Fetal heart rate patterns at 20 to 24 weeks gestation as recorded by fetal electrocardiography*. J Mattern Fetal Neonatal Med, Early Online 1 – 5
2. Matthew O’Connor MD, Nancy McDaniel MD, William J. Brady MD. *The pediatric electrocardiogram part III: Congenital heart disease and other cardiac syndromes.* American Journal of Emergency Medicine (2008) 26; 497-503
3. Fanaroff and Martin’s *Neonatal – Perinatal Medicine, Diseases of the Fetus and Infant*. 8^th^ Edition, 2006. Mosby Elsevier. Page 1218 – 1219

# 13. APPENDIX I

| Congenital defect | Per 1000 live births |
| --- | --- |
| Transposition of the great vessels | 0,59 |
| Fallot’s tetralogy | 0,31 |
| Ventricular septal defect | 2,88 |
| Atrial septal defect | 1,14 |
| Pulmonary stenosis/atresia | 0,75 |
| Aortic stenosis | 0,25 |
| Hypoplastic left heart | 0,22 |
| Coarctation aorta | 0,49 |

# Table 1. Reefhuis J, Samrén EB, Diem MT van. Tables 1981 – 1998. EUROCAT registration of congenital anomalies Northern Netherlands and Southwestern Netherlands. Groningen: University of Groningen, 2000.

# 14. APPENDIX II

| **Congenital defects** | **ECG Findings** |
| --- | --- |
| **Aortic stenosis** | Left ventricle hypertrophy |
| **Atrial septal defect**  **Primum type**  **Secundum type** | Left anterior hemiblock  First-degree AV block  Right axis deviation |
| **Coarctation of the aorta** | Right bundle branch block  Right ventricle hypertrophy |
| **Mitral stenosis** | Right axis deviation  Right ventricle hypertrophy  Right atrial hypertrophy  Left atrial hypertrophy |
| **Pulmonary atresia** | Left ventricle hypertrophy |
| **Pulmonary stenosis** | Right ventricle hypertrophy |
| **Fallot’s Tetralogy** | Right axis deviation |
| **Transposition of the great arteries (complete transposition)**  **Intact ventricular septum**  **VSD and/or PS** | Right atrial hypertrophy  Right ventricle hypertrophy  Right atrial hypertrophy  Biventricular hypertrophy,  right atrial hypertrophy,  bi-atrial hypertrophy |
| **Transposition of the great arteries**  **(“corrected transposition”)** | AV block, 1^st^ to 3^rd^ degree  Atrial arrhythmias (SVT, atrial fibrillation)  Left atrial hypertrophy or bi-atrial hypertrophy |
| **Tricuspid atresia** | Left anterior hemiblock  Left ventricle hypertrophy  Right atrial hypertrophy |
| **Ventricular septal defect**  **Small shunt**  **Moderate shunt**  **Large shunt**  **Pulmonary vascular obstructive**  **disease (Eisenmenger’s syndrome)** | Normal  Left ventricle hypertrophy, left atrial hypertrophy  Biventricular hypertrophy, left atrial hypertrophy  Right ventricle hypertrophy |

Table 2. Myung K. Park, MD; Warren G. Guntheroth, MD. How to read pediatric ECGs. Page 250-251
