# Supplementary material for: The electrical heart axis of the fetus between 18 and 24 weeks of gestation: A cohort study
Source: PLoS One. 2021 Dec 16;16(12):e0256115. doi: 10.1371/journal.pone.0256115 (PMC8675734; doi:10.1371/journal.pone.0256115)
Supplement: Attachment — Submitted filename: respons to reviewers Plos 24-1.docx [file pone.0256115.s003.docx]

Reviewer #1

1. Line 101. Some CHD may not appear clinically till some months or years later of life (ASD, mild CoA). In this medical examination, do you usually make an echocardiogram to check normal heart?

*No, the check-up consists of auscultation, and physical examination of the child post-partum (3 weeks and 3 months post-partum) by a medical doctor.*

2. Line 113. A 30 minutes test is so long. As authors described previously, axis is a “weapon” to determine (usually) ventricular muscle mass predomination. Don´t authors think thay 30 minutes is enough time to get a four chamber view of the fetus heart?

*We agree that a 30 minutes measurement is long, this time was taken for research reasons only, i.e. to be able to collect more data. When used in practice, we believe this should take no longer than 5-10 minutes.*

3. Authors don´t describe variability intra and interobservator of ECG processing and fetal orientation.

*The data processing is computerized and intra- and interobserver variability will not be an issue. For the fetal orientation, a protocol how to handle the transducer of the ultrasound was used to minimize intra- and interobserver variability.*

4. Line 177. Why authors don´t have fetal orientation of these patients?

*The researchers were dependent on the room provided in the center where they were working that day. In some cases an ultrasound device was not available due to lack of space or when the ultrasound device was needed for urgent clinical matters.*

5. Line 196-200. “The median and interquartile range (IQR) of the ̃ coordinate was 0.347 (1.660) and that of the ̃ coordinate was 0.327 (0.956). Based on these normalized coordinates, the average frontal angle was determined at 122.68° (90% PI: -25.6°; 270.9°)”
As authors show on figure 4, authors describe wide range values of the fetuses heart. I don´t think authors can conclude that 122.68° is normal axis of the fetus heart. Prediction intervals include the most part of the frontal coordinates.

*The sentences have been adapted.*

6. Line 210. As said previously. So wide range prediction intervals should´t let to conclude that 122 is normal axis.

*We have reformulated the manuscript to describe the axis as the mean electrical heart axis, rather than stating that this is the normal electrical heart axis.*

7. Line 233. An screening parameter should have hight sensibility. Axis heart variation from righ axis only allow to diagnose a few CHD.

*This sentence has been adapted.*

8. Line 244. Superior axis shown in AVSD is sometimes between -40 and -150º. Authors PI include this interval (Ref. Park's pediatric cardiology for practitioners, 2014)

*We agree with the reviewer. The wide range of the PI is indeed a limitation of our results, suggesting that the use of the electrical heart axis alone is not good enough for CHD screening. We have stressed this in the revised manuscript.*

9. References. Cites 14-16. A novel method por screening should be supported on recent bibliography. Pregnatal and neonatal diagnosis of CHD have changed substantially from 1950s

*Up to our knowledge there is no recent accessible data regarding the electrical heart axis in fetuses. Progress in the detection of CHD has mostly been made with ultrasonography. Hence, indeed the diagnosis of CHD has improved since the 1950s. But the use of ECG for antenatal CHD screening, the focus of our manuscript is new and therefore no literature exists on this topic. .*

Reviewer #2: The authors have analysed the fetal ECG on mid-term pregnant women (around 20 weeks of gestation) and have measured the average heart electrical axis in the frontal plane.

- The text is highly fragmented, specially the Introduction. More details should be included in this part to follow the reasoning: which scans are made, for example.

*The introduction has been adapted and we believe is now less fragmented and more pleasant to read. The 20 week fetal anomaly scan is the ultrasound examination performed around mid-gestation in most developed countries We have modified the manuscript to make this more clear..*

- Punctuation should be revised along all the text.

*The punctuations has been checked and revised where needed.*

- Line 61: “detection rates depend on the experience of the sonographer, fetal
position and habitus of the mother”. What do authors refer with habitus of the mother? How can they affect to the recording acquisition?

*For ‘habitus of the mother’ one can read ‘BMI of the mother’. The higher the BMI the more difficult it may be for the sonographer to make clear images of the fetus, because the distance to the fetus is greater giving a lower quality of the ultrasound images. We have modified the text accordingly.*

- Introduction: refs [14-16] to previous studies were published in 1955. Are there no more recent references?

*Up to our knowledge there is no recent accessible data regarding the electrical heart axis in fetuses. Progress on detection of CHD has mostly been made with ultrasonography. Hence, indeed the diagnosis of CHD has improved since the 1950s. But the use of ECG for antenatal CHD screening that is presented in our manuscript is new and therefore no literature on this topic exists. .*

- There is also excessive auto-citation of the authors: I’ve seen at least 14 references out of 32.

*10 out of 32 references are indeed auto-citations. Most of the auto-citations are used to refer to more detailed work that has been published earlier for readers who which to have more detailed information.*

*To reduce the number of auto-citations, we have omitted a few of them.*

- My main concern is: what is the original work presented by authors in this paper and which is the improvement with respect to the current state of the art? In my opinion, there are 2 main problems:
1.- Authors just give an extremely wide range of the electrical heart axis for control fetus. In fact, they say “The wide prediction interval for the frontal heart axis found in our cohort, is unfavorable for future implementation of this method for screening purposes”. And it really is. In addition, authors do not compare with the ECG of fetus with CHD, they just say the wide range for controls. So, what is the main finding of the paper?

*The aim of this paper is to define reference values for the electrical heart axis of healthy fetuses in mid-pregnancy. This has never been done before. This paper shows the reference values, with the prediction intervals. Unfortunately, these prediction intervals are wide and therefore may not be suitable for screening for CHD. But we do believe that it is important data to publish to help future research. For instance, our findings might motivate the research towards other ECG characteristics that can complement the heart axis and ultimately provide better screening of CHD. We further elaborate on this issue in the discussion section of this manuscript.*

2.- What is the main novelty? Authors have published “Normal ranges for fetal electrocardiogram values for the healthy fetus of 18-24 weeks of gestation: a prospective cohort study” and “The standardized 12-lead fetal electrocardiogram of the healthy fetus in mid-pregnancy: A cross-sectional study” with the same cohort under study.

*The earlier work that the reviewer refers to is the study protocol (ref 21) and the manuscript that describes the method for calculating a fetal ECG that standardizes for fetal position (ref 22). This manuscript shows the electrical heart axis in the study population and how we were able to define reference values for the electrical heart axis in mid-pregnancy. In other words, the current paper describes the clinical results of applying the methods and protocols described before.*

- Methods section should also be improved and including more details, in case results worth it.

*We have modified the methods section and have included more detail on the measurement protocol. Detailed information on the signal processing of the ECG is described in the literature. We believe that this paper would mostly attract readers with a more clinical background than engineering background. The former group would be mostly distracted from detailed information on signal processing. The latter group is referred to detailed information on the signal processing, making the current manuscript less fragment and less cluttered.*

- “Studies in neonates with CHD have already shown the relevance of the electrical heart axis in certain types of CHD. [14–16]”. But no comparison is included.

*The sentence had been adapted to: Studies in neonates with CHD have already shown abnormal electrical heart axes in certain types of CHD.
The paragraph that comes after this statement includes multiple examples of abnormal electrical heart axis in CHD.*

Reviewer #3: On the 328 fetal ECGs performed between 18 and 4 weeks of gestation authors shows that the average electrical heart axis of healthy fetuses around mid-gestation is oriented to the right, which is, in line with muscle distribution in the fetal heart. the average frontal angle was determined at 122.68° (90% 198 PI: -25.6°; 270.9°).

The article is very well written and very clear, the methodology is very well explained.
Conclusion is missing in the Abstract

*We thank the reviewer for his/ her positive comments.*

*The abstract has been adapted and a conclusion has been added.*

As several pitfalls to this new process are being discussed :

- The current difficulty of performing n on-invasive fetal electrocardiography and the time it takes for a patient to do so
 *We agree that a 30 minutes measurement is long, this time was taken for research reasons only to be able to collect more data. When used in practice, we believe this should take no longer than 5-10 minutes.*

- The very wide confidence interval results for electrical heart axis : 122.68° (90% 198 PI: -25.6°; 270.9°).

*We agree with the reviewer. The wide range of the PI is indeed a limitation of our results, suggesting that the use of the electrical heart axis alone is not good enough for CHD screening. We have stressed this in the revised manuscript.*

- If we imagine apply it to fetuses with CHD, in my opinion it makes little clinical sense because many CHDs have a normal heart axis so this examination will not detect them. Furthemore, because of the results of this preliminary study with a wide CI of electrical heart axis it is therefore possible that some CHDs have a straight axis and are not detected and on the contrary normal fetuses may have an axis at 90°. I therefore suggested an improvement of the ultrasound scan in the screening of CHID, as in some countries, because EUROCAT shows that this screening is variable according to the country but that it can be higher than 40%.

*Unfortunately, the prediction intervals are wide and therefore may not be suitable for screening for CHD. But we do believe that it is important data to publish to help future research. For instance, our findings might motivate the research towards other ECG characteristics that can complement the heart axis and ultimately provide better screening of CHD. We further elaborate on this issue in the discussion section of this manuscript. We also believe that the fetal ECG will not replace the ultrasound, but may complement the ultrasound. We agree with the reviewer that the ultrasound scan should also be improved by, for example using a protocol to standardize the fetal anomaly scan.*

However, this preliminary study is interesting and well constructed and shows that this study of the fetal electrical heart axis is feasible but still in development and that technical pitfalls still need to be resolved in the years to come. The authors had the merit to show this and it is a huge work.

*We thank the reviewer for his/her kind comments*
