# Supplementary material for: The electrical heart axis of the fetus between 18 and 24 weeks of gestation: A cohort study
Source: PLoS One. 2021 Dec 16;16(12):e0256115. doi: 10.1371/journal.pone.0256115 (PMC8675734; doi:10.1371/journal.pone.0256115)
Supplement: Attachment — Submitted filename: Response-reviewer-4.docx [file pone.0256115.s005.docx]

**AUTHOR’s RESPONSE:**

Reviewer #4: PONE-D-21-02858R1: statistical review

SUMMARY. In this study, the electrical heart axis is proposed as a predictor of healthy fetuses around 20 weeks of gestation. The statistical analysis is based on a sample of frontal angles. Although the methods seem correct and the results are sound, some statistical computations are nonstandard because they belong to circular statistics. As such, these methods deserve a larger description than that one given by this version of the paper: see the specific issues below.

SPECIFIC ISSUES

1. The average frontal axis is a circular mean. Was it computed by the traditional ratio of trigonometric moments? Please clarify.

- Yes, we took the maximum likelihood estimate (mle) of the mean direction parameter $\mu$ of a von Mises distribution as the average frontal axis which is the same as the first sample trigonometric moment: $\bar{\theta}=\text{atan2}(\bar{S}/\bar{C})$, where  $\bar{C}=\frac{1}{n}\sum_{j=1}^{n} \cos\theta_{j}$ and $\bar{S}=\frac{1}{n}\sum_{j=1}^{n} \sin\theta_{j}$. The praragraph has been adjusted in the manuscript (lines 176-179).

2. It seems that the confidence interval of the average frontal axis has been computed by assuming that the data are sampled from a von Mises distribution. Please provide the value of the concentration parameter of the von Mises distribution that has been used for computing the confidence interval.

3. Please clarify whether the von Mises parameters have been estimated by maximum likelihood methods. In this case, please clarify if the bias of the maximum likelihood estimate of the concentration parameter has been corrected or not.

- We would like to answer the reviewer’s comments 2-3 together since they are related to each other. First, we would like to mention that we computed the prediction interval for the frontal axis rather than the confidence interval. Nevertheless, reviewer 4’s comments regarding the bias of the concentration parameter $\kappa$ are still valid. Indeed, we used the mle of the $\kappa$ without bias corrections for the calculation of the prediction interval. Albeit the fact that the correction method proposed by Best and Fisher (1981) is still biased for small values of $\kappa$ when the sample size is not large, we were able to verify that prediction interval based on the uncorrected $\hat{\kappa}$ has close-to-nominal coverage probability via simulations (see Table 1 below). In this simulation study, for each simulation run, we drew 281 independent random samples from a von Mises distribution with parameter $\mu=4.142$ and $\kappa=0.526$ (these values are also parameter estimates of the real data which the reviewer requested) and estimated the parameters using maximum likelihood. Based on the estimated parameters, we computed the corresponding quantiles of the von Mises distribution as our prediction interval. Thereafter, an independent sample was again drawn from the same distribution as a “future” observation. Across 10000 simulation runs, we calculated how often the prediction intervals contained the “future” observations.

| Prediction interval | Coverage probabilities |
| --- | --- |
| 99% | 98.95% |
| 98% | 98.02% |
| 97% | 96.72% |
| 96% | 96.06% |
| 95% | 95.11% |
| 94% | 93.98% |
| 93% | 93.05% |
| 92% | 91.46% |
| 91% | 90.37% |
| 90% | 90.33% |

4. The von Mises density is just one of the possible distributions that one can choose for circular data modelling. Without asking for a formal test of goodness of fit, could the authors at least overlap the estimated density on the empirical rose diagram of the data? This will show whether the von Mises provides a reasonable fit in this case study.

- We agree with the reviewer that the von Mises distribution is just one of many possible distributions. We hereby provide the plot of the estimated density superimposed on the circular diagram.
